# Mixed-Level Neural Machine Translation

**DOI:** 10.1155/2020/8859452

**Published:** 2020-11-29

**Authors:** Thien Nguyen, Huu Nguyen, Phuoc Tran

**Affiliations:** ^1^Faculty of Information Technology, Ton Duc Thang University, Ho Chi Minh City, Vietnam; ^2^Faculty of Information Technology, Ho Chi Minh City University of Food Industry, Ho Chi Minh City, Vietnam; ^3^Natural Language Processing and Knowledge Discovery Laboratory, Faculty of Information Technology, Ton Duc Thang University, Ho Chi Minh City, Vietnam

## Abstract

Building the first Russian-Vietnamese neural machine translation system, we faced the problem of choosing a translation unit system on which source and target embeddings are based. Available homogeneous translation unit systems with the same translation unit on the source and target sides do not perfectly suit the investigated language pair. To solve the problem, in this paper, we propose a novel heterogeneous translation unit system, considering linguistic characteristics of the synthetic Russian language and the analytic Vietnamese language. Specifically, we decrease the embedding level on the source side by splitting token into subtokens and increase the embedding level on the target side by merging neighboring tokens into supertoken. The experiment results show that the proposed heterogeneous system improves over the existing best homogeneous Russian-Vietnamese translation system by 1.17 BLEU. Our approach could be applied to building translation bots for language pairs with different linguistic characteristics.

## 1. Introduction

No researchers have addressed the problem of neural machine translation for the Russian-Vietnamese language pair. The primary aim of our work is to study neural machine translation for this language pair. Therefore, we attempt to build and analyze Russian-Vietnamese neural machine translation systems. One of the first problems we faced when building a Russian-Vietnamese neural machine translation (NMT) system is to choose a suitable embedding level. There are different translation unit systems, on which the embedding vectors are based. In this work, we use two terminology systems of the translation unit from the technical and linguistic points of view. Technically, we have subtoken, token, and supertoken in increasing order of size. Technical terminology is applied for both Russian source and Vietnamese target languages. Meanwhile, linguistic classifications are different for these languages. In the Russian language, we have subword and word. In the Vietnamese language, we have subsyllable, syllable, and word. The correspondence between technical and linguistic terminology is presented in Table 1.

Initially, researchers [1–4] used tokens as translation units in NMT models. Technically, a token is a sequence of characters delimited by spaces or a punctuation. Linguistically, a token may be a word or a syllable depending on the language. For example, Russian tokens or English tokens are words, and meanwhile Vietnamese tokens are syllables. In NMT models, a sequence of source tokens is passed through the embedding layer to become a sequence of source numerical vectors. The source numerical vectors are then transformed by the NMT models to target numerical vectors. The NMT models then use the sequence of target numerical vectors to predict a sequence of target tokens. The main shortfall of the token-based NMT models is that they cannot handle out-of-vocabulary and rare-token problems, especially in cases of low-resource language pairs, such as Russian-Vietnamese.

Many attempts have been made to tackle the out-of-vocabulary and rare-token problems. Some authors [5–9] proposed to use subtokens instead of tokens as the translation unit, and some other authors [10–13] even took extreme approaches splitting tokens into smallest character-based subtokens. Due to computational capacity limitation, we could not build character-based NMT models. Since Russian tokens are lengthy, consisting of multiple characters, dividing Russian tokens into separated characters leads to over lengthy sentences. However, computational requirement for building NMT systems to handle lengthy sentences is very high. Lee et al. [10] claimed that they trained their character-based NMT model for approximately two weeks. We were unable to meet such huge computational requirement; therefore, we did not apply the character-based translation unit system in building our NMT system.

After searching for a suitable translation unit system, we have noted a common characteristic of available translation unit systems. In those systems, the translation unit on the target side is the same as the translation unit on the source side. NMT models translate from tokens to tokens or from subtokens to subtokens. Our preliminary experiment of translating from Russian to Vietnamese shows that NMT systems are homogeneous in terms of the translation unit, in other words, applying the same translation unit on both source and target sides may not be the best solution. Given that homogeneous NMT systems have not satisfied our requirement, we propose a novel mixed-level NMT model instead. Specifically, in source sentences, we use subtokens as the translation unit, while in target sentences, we use supertokens as the translation unit.

The rest of this paper is divided into four sections.  Section 2 gives a brief overview of related works. A novel mixed-level NMT model based on the state-of-the-art transformer model for the Russian-Vietnamese language pair is described in Section 3.  Section 4 analyses experiment results. Our conclusions are given in the final section.

## 2. Related Works

In this section, we briefly describe different translation unit systems in NMT models which directly influence our study.

Luong and Manning [14] tokenized English source sentences and Vietnamese target sentences with the default Moses tokenizer [15] and fed these tokens into an NMT system. Linguistically, a token in English is a word, and a token in Vietnamese is a syllable, considering that in Vietnamese sentences, whitespace characters separate not words but syllables, as stated by Oh et al. [16]. In this work, we also use a similar translation unit system translating from Russian tokens to Vietnamese tokens and consider it as the baseline token-to-token system. Linguistically, we tokenize Vietnamese sentences into syllables and tokenize Russian sentences into words since Russian words, just like English, are separated by whitespace characters.

Sennrich et al. [5] developed a new subtoken-based NMT system for translating between Russian and English. They proposed to segment both source and target tokens into subtokens and used these subtokens as translation units. Their method is called byte-pair encoding (BPE). Firstly, the BPE method considers tokens as sequences of characters and adds the characters to the symbol dictionary. Next, the BPE method identifies the most frequent symbol pairs and merges them. The merged symbol pairs are then added to the symbol dictionary. The process is repeated until a predefined number of merge operations is reached. For example, the Russian token “первоначальную” (meaning: initial) is divided into two subtokens “первонача@@” and “льную.” Characters “@@” are added to a subtoken to inform that the subtoken is not the ending part of any original token. In the BPE method, the merge operations learned in the training dataset can be applied to test sentences. Sennrich et al. used the BPE method to segment out-of-vocabulary test tokens into in-vocabulary subtokens in respect to the training dataset. The authors reported a significant performance improvement when translating between Russian and English. The BPE method was also applied for both source and target languages in [17, 18] to improve translation from English to Vietnamese. In this work, we also experiment the BPE method for translating from Russian to Vietnamese.

Kudo [7] proposed an alternative approach to segment tokens into subtokens according to the unigram language model (UNI). The UNI method considers a sentence as a sequence of subtokens. Subtokens are supposed to appear independently; therefore, the probability of a sequence is the product of the subtoken probabilities. The chosen subtoken sequence for a sentence is the sequence with the largest probability in respect to all possible sequences from that sentence. An example of segmentation by the UNI method is as follows. The Russian token “первоначальную” (meaning: initial) is divided into two subtokens “_первоначальн” and “ую.” In contrast to the convention in the BPE method, in the UNI method, an underscore character “_” is added to a subtoken to inform that the subtoken is the beginning part of an original token. As in the BPE method, character “_” is introduced to reconstruct the original token from its subtokens. In our preliminary experiment on studying Russian-Vietnamese machine translation, we notice that the UNI method does not improve translation quality over the BPE method; therefore, in this work, we only use the BPE method for splitting tokens into subtokens.

## 3. Mixed-Level Model for Russian-Vietnamese Neural Machine Translation

Linguistically having studied subtokens split from Russian and Vietnamese tokens with the BPE method, we have discovered different effects. On the Russian side, subtokens have some meanings. For example, the Russian token “первоначальную” (meaning: initial) is divided into two subtokens “первонача@@” and “льную,” and the subtoken “первонача@@” is the root part of many other tokens related to something initial, such as первоначать, первоначальный, первоначальные, первоначальная, and первоначальным, while the subtoken “льную” has a grammatical role of adjective of the feminine gender in accusative case. Russian is a morphologically rich language. A token is a surface form of a lemma. A surface form is generated from a lemma according to its grammatical role in the sentence. Linguistically, the BPE method successfully divides Russian tokens into meaningful subtokens. On the other hand, subtokens which are divided from Vietnamese tokens are not informative. For example, the Vietnamese token “nghiệt” is divided into two subtokens “ngh@@” and “iệt.” We have not found meanings of these subtokens. Perhaps, it is due to the fact that Vietnamese is an analytic language which does not utilize inflections as a grammatical tool. Collectively, splitting tokens into subtokens is useful on the source Russian side and meaningless on the target Vietnamese side. Furthermore, a lot of Vietnamese tokens are homophones with the same written form but different meanings. For example, the Vietnamese token “dai” has many senses (age, big, packet, replace, replacement, representative, and represent). Representing a Vietnamese sentence as a sequence of tokens leads to sense ambiguation of token-based embeddings, consequently, negatively affects translation quality. To achieve sense disambiguation, we propose to use supertokens instead of tokens as the translation unit on the target side. Supertokens are formed in the result of linguistically merging neighboring tokens. In fact, Vietnamese tokens are syllables, so we merge them to form supertokens which are compound words in the linguistic point of view. Taken it all together, we propose a mixed-level model for Russian-Vietnamese NMT. On the source side of translation, we split Russian tokens into subtokens. On the target side of translation, we merge Vietnamese tokens into supertokens. In brief, we apply opposite operations in segmenting sentences into sequences on the source and target sides.

In this work, we use the state-of-the-art NMT model called transformer [19]. The transformer model follows the common pattern of sequence to sequence models.

The step-by-step procedure of the mixed-level transformer-based NMT model for the Russian-Vietnamese language pair is presented in Algorithm 1.

## 4. Materials

Our parallel corpus consists of 33,027 Russian-Vietnamese sentence pairs. First, we chose the special-character-free Russian sentences with length from 10 to 19 from news.

Commentary data (Download at: http://www.statmt.org/wmt13/training-parallel-nc-v8.tgz) of shared task are from the Machine Translation of ACL 2013 Eighth Workshop on Statistical Machine Translation. Having chosen the Russian sentences, we then applied the Google translate service to translate them into Vietnamese. After that, we manually corrected these initial Vietnamese sentences, making them sound naturally and keeping the meaning of the Russian source sentences. Finally, we randomly divided our parallel corpus into three datasets: training, development, and testing with the sizes 30027, 1500, and 1500, respectively, following the design pattern of datasets used in the studies [20, 21] for the low-resource Chinese-Vietnamese language pair. Specifically, we randomly took 1500 sentence pairs to create the testing dataset and another 1500 sentence pairs for the development dataset. The remaining 30027 sentence pairs in the parallel corpus comprises the training dataset. The summary of the datasets is presented in Table 2.

## 5. Methods

To evaluate the proposed mixed-level model in comparison with other models, we performed six experiments. The first four columns of Table 3 show the setup of the experiments. In each experiment, we applied a translation unit on the source side and another translation unit on the target side, when building a corresponding NMT model. Translation unit on the Russian source side can be token and subtoken. Translation unit on the Vietnamese target side can be subtoken, token, or supertoken. For instance, the baseline token2token model has token as the translation unit on the source side and token as the translation unit on the target side.

In experiments, each model has its own translation component. In this work, we deployed transformer models as translation components. We based our transformer implementation on the work of Ben Trevett (Download at: https://github.com/bentrevett/pytorch-seq2seq) with the hyperparameter values presented in Table 4. To train models, we used the training dataset. To prepare the compatible input/output for each model, we did preprocessing steps on the training dataset. Source/target tokens are prepared with the default Moses tokenizer [15]. In our systems, we do not filter out stop works or punctuation marks. All of them are considered as tokens. The systems treat them the same way as other tokens in translation. We believe that these punctuation marks and stop words contain information though not as important as other tokens. Vietnamese target supertokens are constructed from tokens by the VnCoreNLP tool provided by Vu et al. [22]. Unlike whitespaces in English separating words, whitespaces in written Vietnamese delimit syllables. The VnCoreNLP tool uses a rule-based learning model to concatenate neighboring syllables to words, constructing supertokens from tokens. Subtokens on both Russian source side and Vietnamese target side are segmented from tokens with the BPE method [5]. First, the BPE method considers tokens as sequences of characters. Then, the BPE method identifies the most frequent character pair and merges all its occurrences in the training dataset. This step of identifying and merging is repeated 10000 times for the Russian source side and 1500 times for the Vietnamese target side of the dataset. The merging operations learned in the training dataset are then applied to the development and testing datasets.  Table 5 shows results of the preprocessing step on a sentence pair in the training dataset. Here, to denote the boundary between items in sequence, we use the character “*|*.”

We optimized parameters of each NMT model with 20 epochs of the training dataset using the Adam optimizer as the one proposed by Devlin et al. [23]. Each time we passed through an epoch, we saved the values of model parameters. Finally, we selected the one with the least lost value in the development dataset.

We assessed the models with the testing dataset. Each Russian-Vietnamese sentence pair in the testing dataset is used as follows. The Russian sentence is transformed into a sequence of subtokens/tokens, satisfying the requirement of an NMT model. After that, the NMT model processes the source sequence and returns a target sequence of subtoken/token/supertokens according to the target translation unit of the NMT model. The target sequence is then postprocessed to form the predicted Vietnamese sentence. The postprocessing step depends on the item type of the target sequence. If the item type is token, then target tokens are concatenated with the whitespaces between them to form a Vietnamese sentence. If the item type is subtoken, first we merge subtokens ended with characters “@@” with immediately following subtokens to form tokens, and then we concatenate resulting tokens to form a Vietnamese sentence. If the item type is supertoken, first we divide supertokens into tokens by replacing characters “_” with whitespace characters, and then we apply token concatenation to form a Vietnamese sentence. After translating all Russian sentences in the testing dataset, we evaluate the predicted Vietnamese sentences with the lowercase BLEU score–a measure of similarity between them and the corresponding reference Vietnamese sentences in the testing dataset. We used the natural language toolkit NLTK [24] to calculate the BLEU score.

## 6. Results and Analysis

The BLEU scores of translation results by the models are presented in Table 3. As expected, we observe the tendency that on the Russian source side, subtoken is favorable to token as the translation unit. The average BLEU score by NMT models with token as the translation unit on the Russian source side is (34.45 + 33.44 + 31.50)/4 = 33.13 BLEU. Meanwhile, the average BLEU score by NMT models with subtoken as the translation unit on the Russian source side is superior with the value (38.16 + 37.23 + 36.99)/3 = 37.46 BLEU.

On the Vietnamese target side, we have found the tendency, opposite to the trend on the source side, that the bigger the size of the translation unit is, the higher the BLEU score is. The average BLEU score (31.50 + 36.99)/2 = 34.245 BLEU by NMT models with target subtoken is less than that by models with target token (33.44 + 37.23)/2 = 35.335. In turn, the average BLEU score by NMT models with the target token is less than that by models with the target supertoken (34.45 + 38.16)/2 = 36.305 BLEU.

The experiment results also highlight the best NMT model. The sub2super model uses subtokens on the source side and supertokens on the target side. Compared to the baseline tok2tok model, the sub2super model improves translation results by 38.16 − 33.44=4.72 BLEU. In addition, the sub2super model outperforms the sub2sub model by 38.16 − 36.99=1.17 BLEU.

In summary, machine judgment on experiment results proves our proposal of reducing the size of the translation unit on the Russian source side and increasing the size of the translation unit on the Vietnamese target side.

We further verified our findings by human judgment on translation results. Some typical translation results are presented here.

First, we look at translation results of a short Russian sentence (see Table 6). The translation result by the baseline tok2tok model contains some good word translations, such as “marx” for “маркса.” But in general, the meaning of the translation is wrong. Meanwhile, the sub2sub model misses the word “карла” (Karl) and adds unwanted phrase “sắt màn” to the translation result. The sub2super model gives a wrong translation “đã Viết bài Viết bài <unk>” for the word “карла” (Karl). Although the two last models do mistranslate some words, they succeed in translating the main idea. The meaning of translation results by sub2sub and sub2super models is similar to the meaning of the Vietnamese gold reference.

Next, we assess NMT models with a longer Russian sentence (see Table 7). The length of translation results by the NMT models is comparable with the length of the Vietnamese gold reference. All NMT models are successful in translating most words, but the sub2sub model gives a wrong overall meaning of translation by mistranslating key phrase “отчаянно желают” (are desperate to). Translation results by the baseline tok2tok and sub2super models are better in conveying the meaning of the source sentence. The sub2super model gives a translation meaning which is closest to the Vietnamese gold reference. Specifically, the sub2super model translates the phrase “отчаянно желают” into rất muốn (really want), while the baseline tok2tok model translates that phrase into muốn (really want).

Lastly, we evaluate NMT models with another long Russian sentence (see Table 8). All NMT models give predicted sentences longer than the Vietnamese gold reference. Although the translation result by the sub2super model is not perfect, it is far better than the translation results by the baseline tok2tok and sub2sub models. The former model adds an unnecessary phrase “buộc phải chịu sự thiên vị cho các biện pháp truyền thông nói chung,” but we can understand the general idea of translation. Meanwhile, translation results by the latter two models are incomprehensible.

Taken together, human judgment on translation results is in agreement with machine judgment.

Following both machine and human judgments on translation results, we can state that the sub2super model is the most appropriate for translating from Russian to Vietnamese, and our proposal to reduce the translation unit on the Russian source side and increase the translation unit on the Vietnamese target side is a clear improvement on the current homogeneous translation unit systems.

## 7. Conclusions

In this article, we have analyzed different translation unit systems for Russian-Vietnamese neural machine translation. Based on our linguistic understanding of morphologically rich Russian language and analytic noninflectional Vietnamese language, we propose a novel mixed-level model for translating from Russian to Vietnamese. The mixed-level model uses subtokens as the input and supertokens as the output. Experiment results prove our proposal with a better BLEU score for the model. Knowing the limit of machine judgment in the form of the BLEU score, we have thoroughly studied the translation results with human judgment. The analysis by human judgment is essentially the same as the analysis by machine judgment. Considering the results of both machine and human judgments, we conclude that the best NMT model for the Russian-Vietnamese language pair is mixed level.

In addition, we believe that our proposal of the mixed-level NMT model is not only useful for translating from Russian to Vietnamese but also applicable for building translation bots for other language pairs where the source side is a morphologically rich language and the target side is an analytic noninflectional language, such as Ukrainian-Vietnamese, Czech-Vietnamese, German-Vietnamese, and, to some extent, English-Vietnamese.

## Figures and Tables

**Algorithm 1:**
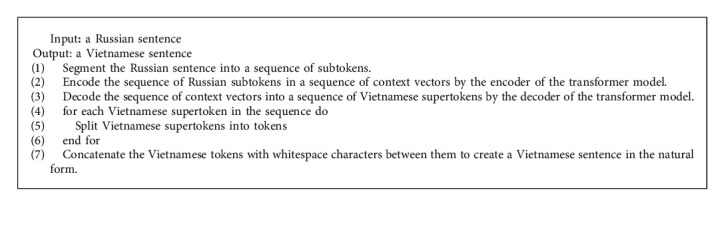
: Mixed-level transformer-based NMT model for the Russian-Vietnamese language pair.

**Table 1:** Correspondence between technical and linguistic terminology of the translation unit.

Technical term	Linguistic term for Russian	Linguistic term for Vietnamese
Subtoken	Subword	Subsyllable
Token	Word	Syllable
Supertoken	None	Word

**Table 2:** Summary of the experiment corpus.

Number of	Russian			Vietnamese		
	Training	Development	Testing	Training	Development	Testing
Sentences	30,027	1,500	1,500	30,027	1,500	1,500
Tokens	438,875	21,820	21,941	693,681	34,436	34,651
Tokens per sentence	14.6	14.5	14.6	23.1	23.0	23.1
Unique tokens	46,789	7,520	7,450	5,402	1,985	2,058

**Table 3:** The BLEU scores of translation results.

Experiment	Model name	Translation unit on the source side (technical*|*linguistic)	Translation unit on the target side (technical*|*linguistic)	BLEU score
1	tok2super	Token*|*word	Supertoken*|*word	34.45
2	tok2tok	Token*|*word	Token*|*syllable	**33.44**
3	tok2sub	Token*|*word	Subtoken*|*subsyllable	31.50
4	sub2super	Subtoken*|*subword	Supertoken*|*word	**38.16**
5	sub2tok	Subtoken*|*subword	Token*|*syllable	37.23
6	sub2sub	Subtoken*|*subword	Subtoken*|*subsyllable	**36.99**

**Table 4:** Hyperparameter values of NMT models.

Hyperparameter	Value
Dictionary	Tokens with occurrence frequency ≥ 2
Dimension of context vectors	256
Number of encoder sublayers	3
Number of decoder sublayers	3
Multihead attention	8 heads
Dimension of encoder feedforward layer	512
Dimension of decoder feedforward layer	512
Dropout level	0.1
Optimizer	Adam
Learning rate	5*e*^−4^
Number of epochs	20

**Table 5:** Input/output of NMT models.

Type	Example
Source sentence	сотрудничество с гаагой, кажется, приносит только боль, унижение и позор.
Target sentence	hợp tác với the hague dường như chỉ mang lại nỗi đau, sự sỉ nhục và xấu hổ.
Sequence of source subtokens	отрудничество|с|гаа@@|гой|,|кажется|,|приносит| только|боль|,|уни@@|жение|и|по@@|зор|.
Sequence of source tokens	сотрудничество|с|гаагой|,|кажется|,|приносит| только|боль|,|унижение|и|позор|.
Sequence of target subtokens	hợp|tác|với|th@@|e|ha@@|gu@@|e|dường|như|chỉ|mang| lại|nỗi|đau|,|sự|s@@|ỉ|nh@@|ục|và|xấu|h@@|ổ|.
Sequence of target tokens	hợp|tác|với|the|hague|dường|như|chỉ|mang|lại|nỗi| đau|,|sự|sỉ|nhục|và|xấu|hổ|.
Sequence of target supertokens	hợp_tác|với|the|hague|dường_như|chỉ|mang|lại|nỗi| đau|,|sự|sỉ_nhục|và|xấu_hổ|.

**Table 6:** Translations from a short Russian sentence.

Tag	Content
Russian	“я не верю в железные законы истории карла маркса.”
Meaning	“I do not believe in the iron laws of the history of Karl Marx.”
Reference	“tôi không tin vào các qui luật lịch sử sắt của karl marx.”
tok2tok	“tôi không tin tức cho luật sư của marx đã gặp rắc rối lịch sử.”
sub2sub	“tôi không tin vào sắt màn luật sắt trong lịch sử của marx.”
sub2super	“tôi không tin vào luật sắt lịch sử của marx đã Viết bài Viết bài <unk>”

**Table 7:** Translations from a long Russian sentence.

Tag	Content
Russian	“в то же самое время египет и саудовская аравия отчаянно желают избежать падения режима ассада.”
Meaning	“at the same time, Egypt and Saudi Arabia are desperate to avoid the collapse of the Assad regime.”
Reference	“đồng thời, ai cập và ả rập xê út đang tuyệt vọng để tránh sự sụp đổ của chế độ assad.”
tok2tok	“đồng thời, ai cập và ả rập saudi muốn tránh sự sụp đổ của chế độ assad.”
sub2sub	“đồng thời, ai cập và ả rập saudi có sẵn sàng tránh chế độ assad.”
sub2super	“đồng thời, ai cập và ả rập saudi rất muốn tránh sự thất bại của chế độ assad.”

**Table 8:** Translations of different lengths from a long Russian sentence.

Tag	Content
Russian	“вместо того чтобы сфокусироваться только на кризисе, они вынуждены нервничать под прицелом телекамер сми.”
Meaning	“instead of focusing only on the crisis, they are forced to get nervous at the sight of media cameras.”
Reference	“thay vì chỉ tập trung vào cuộc khủng hoảng, họ buộc phải lo lắng khi nhìn thấy các camera truyền thông.”
tok2tok	“thay vì cuộc khủng hoảng, chỉ có cuộc khủng hoảng, họ bị buộc phải bị buộc bởi các phương tiện truyền thông bên ngoài truyền thông.”
sub2sub	“thay vì tập trung vào cuộc khủng hoảng, họ buộc phải buộc phải suy dinh dưỡng các biện pháp truyền thông.”
sub2super	“thay vì tập trung vào cuộc khủng hoảng, họ chỉ tập trung vào các biện pháp truyền thông, buộc phải chịu sự thiên vị cho các biện pháp truyền thông nói chung.”

## Data Availability

Our Russian-Vietnamese parallel corpus is available on request. If you are interested in the corpus or you need it to verify our findings, please contact the corresponding author Thien Nguyen via the e-mail address: nguyenchithien@tdtu.edu.vn.
